# Bone and Cartilage Biology

**DOI:** 10.3390/ijms24065264

**Published:** 2023-03-09

**Authors:** Riko Nishimura

**Affiliations:** Department of Molecular & Cellular Biochemistry, Osaka University Graduate School of Dentistry, 1-8 Yamdaoka, Suita, Osaka 565-0871, Japan; nishimura.riko.dent@osaka-u.ac.jp; Tel.: +81-6-6879-2887; Fax: +81-6-6879-2890

Recent technical and conceptual advances in molecular and cellular biology have dramatically advanced bone and cartilage biology. This progress has also improved our understanding of the molecular pathogenesis of bone and cartilage diseases, including osteoporosis, osteoarthritis, and rheumatoid arthritis. This Special Issue aims to provide novel notions for skeletal development, and bone and cartilage diseases.

Toshihisa Komori comprehensively reviewed the roles of Runt-related transcription factor 2 (Runx2) in skeletal development. Runx2, which is a critical transcription factor for bone and cartilage development, regulates the proliferation and differentiation of osteoblasts and chondrocytes by regulating the expression of Col1a1, Col1a2, secreted phosphoprotein 1 (Spp1), integrin-binding sialoprotein (Ibsp), bone gamma carboxyglutamate protein (Bglap)/Bglap2, Indian hedgehog (Ihh), fibroblast growth factor (Fgf), Wnt, and parathyroid hormone-related protein (Pthrp). In particular, the requirement of osteocalcin (Bglap/Bglap2) for the alignment of apatite crystals parallel to the collagen fibers was evoked [1]. Hironori Hojo and Shinsuke Ohba reviewed new aspects of Sp7/Osterix, an essential transcription factor for skeletal development. Two modes of action of Sp7 were elucidated during skeletal genesis. As a canonical mode, Sp7 directly binds to the target genes and regulates their expressions. Conversely, Sp7 indirectly interacts with the target genes as a non-canonical mode, through association with distal-less homeobox 5 (Dlx5) and AP-1-related transcription factors. These ideas would contribute to the understanding of the pathogenesis of osteoporosis [2]. Qing Jiang and colleagues investigated the requirements of Runx2 and CBFB, an essential transcriptional partner for Runx2, in several skeletal tissues using Runx2 and Cbfb mutant mice. Interestingly, the dosage of Runx2 and CBFB required is different for the appropriate development of calvaria, limbs, vertebrae, and ribs [3]. Takeshi Moriishi and colleagues examined the precise role of Sp7/Osterix in bone metabolisms using Sp7 transgenic mice. Sp7 overexpression reduced bone volume and impaired the lacunocanalicular network along with upregulation of Sost expression in the mice. This study suggested the unique role of Sp7 in mechanical stress and bone metabolisms [4].

In a review article, Eijiro Jimi and Takenobu Katagiri emphasized the important role of the nuclear factor-κB (NF-κB) signaling, which not only has a function in the immune response, but also in bone metabolism. Current knowledge of the structure of NF-κB family members and NF-κB signaling was also precisely illustrated [5]. Shawn A. Hallett and colleagues reviewed the molecular mechanisms that underlie the cranial base, which is formed by endochondral ossification and responsible for craniofacial development. In addition to PTHrP-Ihh, FGF, Wnt, bone morphogenetic protein (BMP) signaling, and Runx2, FGF receptor1, 2, and 3 are involved in cranial base formation by MAP kinase signaling interaction [6]. Shawn A. Hallett and colleagues elegantly investigated the role of PTHrP in cranial base development using the gene tracing system in mice. PTHrP-positive chondrocytes have no column-forming abilities in synchondrosis, suggesting an unexpected role of PTHrP in cranial base development [7]. Satoshi Kubota and colleagues reviewed the network system formed by the cellular communication network factors (CCN) family and FGFs during cartilage development. Among the CCN family members, CCN2 associates with FGFs and modulates the function of FGF receptors. Additionally, FGF signaling conducts CCN2 expression and consequently controls chondrocyte metabolisms [8].

Tomohiko Murakami and colleagues provided a novel concept of osteoimmunology in their review article, in which the NOD-like receptor family pyrin domain-containing 3 (NLRP3) inflammasome is involved in bone and joint diseases, such as osteoarthritis. The formation and activation of NLRP3 inflammasome is critical for interleukin-1β production that leads to inflammation and immunogenic responses in bone and cartilage. The potential role of other types of inflammasomes, known as the NLRC4 inflammasome and AIM2 inflammasome, was described [9]. Kridtapat Sirisereephap and colleagues illustrated the recent understanding of osteoimmunology in periodontitis. Considering the prevention of the onset and progression of periodontitis, it is an important key issue to prevent bone resorption by osteoclasts. Immune cells stimulated by initial periodontitis promote the expression of the receptor activator of nuclear factor kappa-B ligand (RANKL) in activated periodontal ligament cells and promote osteoclastic bone resorption. They also described new molecules involved in the activation of inflammation and bone resorption in periodontal tissues [10]. Takuma Matsubara and colleagues evoked the structure and functional role of c-Src tyrosine kinase in osteoclast biology. c-Src is an essential kinase for the function and activity of osteoclast. c-Src interacts with several molecules, including FAK, p130Cas, Pyk2, and cortactin, and regulates ruffled border formation necessary for osteoclastic bone resorption. c-Src has two forms; active and inactive forms are controlled by the phosphorylation of c-Src terminal tyrosine residue. Csk and Cbp are responsible for the regulation of c-Src function through the phosphorylation site [11].

Tomoka Hasegawa and colleagues described the mechanisms of matrix vesicle-mediated mineralization, which is necessary for both intramembranous and endochondral ossification. As a new aspect for osteoblasts and osteocytes, the mechanisms of the accumulation of Ca2+ and PO43− in the matrix vesicles were shown with morphogenetic evidence [12]. Maria Peshkova and colleagues described the potential cartilage tissue regeneration using new cell-free techniques. They proposed a suppression system for inflammation, appropriately using scaffolds, extracellular vesicles, and nano-carriers [13]. Rina Iwamoto and colleagues described positive and negative regulators of Sclerostin expression. Sclerostin inhibits Wnt/β-catenin signaling by binding to the Wnt co-receptor Lrp5/6 and suppressing its bone-forming activity. Sclerostin is produced and secreted in osteocytes. PTH, an anabolic reagent for bone formation, suppresses Sclerostin production by osteocytes by controlling HDAC4 and 5 [14].

To define cell populations within non-degenerating and degenerating human intervertebral discs, Hosni Cherif and colleagues performed single-cell RNA-seq analyses and discovered new biomarkers of intervertebral disc degeneration. These biomarkers would be useful for the diagnosis and development of therapeutic strategies for disc diseases [15]. Lourdes Alcaide-Ruggiero and colleagues discussed the role of several types of collagens in cartilage repair. They also described the fashion of collagen fibril in cartilage [16]. Huan Yu and colleagues illustrated the role of several factors, including nerve growth factor (NGF), calcitonin gene-related peptide (CGRP), C–C motif chemokine ligands 2 (CCL2), tumor necrosis factor α (TNF-α), IL-1β, and Wnt, in osteoarthritis pain [17]. Candide Alioli and colleagues described the expression pattern of lysophosphatidic acids (LPAs), such as LPA1, 2, 3, 4, 5 and 6, during the differentiation of osteoblasts and osteoclasts. Additionally, the role of LPAs in bone metabolism was discussed [18]. Haruhisa Watanabe and colleagues attempted to find biomarkers for temporomandibular joint diseases by focusing on the CCL5-CCR5 axis. The serum levels of CCL5 were significantly increased in temporomandibular joint diseases, but not in a postmenopausal model of ovariectomized rats. The results suggest the specificity and significance of CCL5 in temporomandibular joint diseases [19]. Yvonne Rellmann and colleagues investigated the importance of endoplasmic reticulum (ER) stress in the pathogenesis of osteoarthritis using ERp57 conditional knockout (cKO) mice. ERp57 cKO mice showed an accelerated development of osteoarthritis compared with wild-type mice, suggesting the role of ERp57 in the pathogenesis of osteoarthritis [20]. Marco Ponzetti and colleagues studied the importance of Lipocalin 2 (Lcn2) in Duchenne muscular dystrophy. Lcn2 levels in serum were increased in the model of Duchenne muscular dystrophy. Lcn2 deficiency and treatment with anti-Lcn2 antibodies prevented bone loss and improved muscle health, indicating Lcn2 as a potential therapeutic target for Duchenne muscular dystrophy [21].

I sincerely thank the authors for their contributions to this Special Issue and believe that these notions would help to further our understanding of molecular and cellular mechanisms of bone and cartilage biology, as well as contribute to the development of therapeutic strategies for bone and cartilage diseases.
